# Antimicrobial effect of treating thermoformable cellulose-based materials with essential oils

**DOI:** 10.1007/s00784-026-07111-3

**Published:** 2026-09-16

**Authors:** Lisa Stäuble, Olivier Braissant, Carlalberta Verna, Michael M. Bornstein, Monika Astasov-Frauenhoffer

**Affiliations:** 1 Department of Oral Health & Medicine, University Center for Dental Medicine Basel UZB, University of Basel, Mattenstrasse 40, Basel, 4058 Switzerland; 2 Department of Biomedical Engineering (DBE), Center of Biomechanics and Biocalorimetry, University of Basel, Hegenheimermattweg 167b, Allschwil, 4123 Switzerland; 3 Department of Pediatric Oral Health and Orthodontics, University Center for Dental Medicine Basel UZB, University of Basel, Mattenstrasse 40, Basel, 4058 Switzerland; 4 Department Research, University Center for Dental Medicine Basel UZB, University of Basel, Mattenstrasse 40, Basel, 4058 Switzerland

**Keywords:** Thermoformable, Cellulose, Periodontal, Caries, Essential oil, Antimicrobial

## Abstract

**Objectives:**

To evaluate the antimicrobial effect of essential oil-loaded thermoformable cellulose-based material on the formation of biofilms by *Streptococcus mutans* and three-species biofilms (*Porphyromonas gingivalis*,* Fusobacterium nucleatum and Streptococcus sanguinis*) under in vitro flow conditions.

**Materials and methods:**

Cellulose-based thermoformable materials were pre-treated with either essential oils, or phosphate-buffered saline (control). The materials were combined with saliva-treated hydroxyapatite (HA) discs in a flow chamber system. *S. mutans* and a three-species periodontal biofilm were cultivated under aerobic and anaerobic flow conditions for 16 h. Biofilm formation was quantified using crystal violet staining and antimicrobial activity was assessed via isothermal microcalorimetry (IMC).

**Results:**

Addition of essential oils reduced *S. mutans* biofilm formation by 34% on HA and by 52.5% on cellulose-based material. IMC showed reduced growth rate and total metabolic activity and prolonged lag phase for periodontal pathogens. However, mixed-species periodontal biofilms exhibited minimal changes (-6.3% on HA and + 12% on cellulose-based material).

**Conclusions:**

In-vitro data shows that treating cellulose-based materials with essential oils can significantly reduce the formation of *S. mutans* biofilms, suggesting potential benefits for preventing caries for example during orthodontic therapy by using aligners. However, no significant inhibitory effect was observed against periodontal biofilm, indicating the need for further optimisation of antimicrobial combinations regarding this purpose.

**Clinical relevance:**

The findings provide preclinical evidence that essential oil-loaded thermoformable dental materials reduce biofilm accumulation and provide basis for further evaluation for applications in oral medicine, cariology, and orthodontics. Clinical studies are required to confirm their preventive efficacy and relevance in vivo.

## Introduction

Thermoformed intraoral appliances are widely used in dentistry, including clear aligners and retainers, nightguards and other occlusal splints, as well as bleaching or fluoride trays. Most of these devices are currently fabricated from polymers such as polyethylene, polyester-based terephthalates and ethylene-vinyl acetate, acrylic/PMMA‑based resins and, increasingly, 3D‑printed photopolymers [1–4]. Although clinically proven to be effective for the purposes mentioned, extended daily wear, large unpolished internal surfaces and complex geometries create favorable niches for plaque retention and biofilm formation on both, the appliance surface as well as on adjacent teeth [4]. Increase in supragingival plaque indices [5], shift the oral microbiota toward more cariogenic and periopathogenic profiles, and harbor opportunistic pathogens including *Streptococcus mutans*, *Staphylococcus aureus*, *Enterococcus spp*., *Pseudomonas aeruginosa* and *Candida spp.* [6] Both in vitro and in situ studies demonstrate substantial biofilm formation on conventional splint and retainer materials, with higher roughness of many thermoplastics associated with greater biofilm mass and viable counts, particularly on the inner, tooth‑facing surfaces and marginal areas near the gingiva [4, 6, 7].

In this context, cellulose‑based biomaterials have emerged as sustainable, multifunctional alternatives that can be processed into thermoformable sheets to further be formed into any appliance shape [8]. Recent review by Wodag & Azanaw highlights nanocellulose, cellulose composites and derivatives as eco‑friendly dental materials offering tunable mechanics, good biocompatibility, and the ability to provide controlled drug delivery, antibacterial activity and bioactivity across restorative, adhesive and regenerative applications [9]. Although biofilm formation on dental appliances is strongly influenced by surface morphology, surface chemistry and surface charge, current research is exploring methods of incorporating antimicrobial components into the structure of dental appliances to obtain such antimicrobial dental material, for example, by using mussel-inspired [10] or N-halamine [11] coatings, zinc oxide and magnesium oxide [12] or chitosan nanoparticle [13] coatings or resin containing zwitterionic materials [14]. Among others, essential oils have been considered. As the main component of cinnamon, cinnamaldehyde is known to have antimicrobial, antioxidant, anti-inflammatory, antidiabetic, antitumoral and liquid-lowering properties [15]. It has been shown to be effective against the *S. mutans* and *Porphyromonas gingivalis* that is a known pathogen causing periodontal diseases [16, 17]. Cinnamaldehyde is often associated with other essential oil components, such as limonene or eucalyptol [16, 18]. Both of these oils have also demonstrated antimicrobial activity against *S. mutans* [15, 19, 20]. Recent studies have shown that it is possible to incorporate cinnamaldehyde into thermoformable cellulose-based polymers used for dental appliances, and the impregnation of these polymer compounds showed delayed biofilm formation of oral streptococci, such as *S. mutans* and *S. mitis* on the surface [15, 21]. Moreover, other essential oils, e.g. carvacrol is reported to show bactericidal and antibiofilm activity against oral streptococci and periodontal bacteria [22] and in combination with cinnamaldehyde or antibiotics reveals synergistic effects [23, 24]. However, not all essential oils how high antimicrobial activity, only moderate efficacy has been measured when using limonene or eucalyptol [25]; while data on methyl salicylate and triacetin has been reported in more complex formulations [26].

Unlike strong and high-concentrated mouthwashes or other liquid disinfectants such as chlorhexidine, these materials can function as quasi carrier systems capable of continuous release antimicrobial agents slowly and selectively at the tooth-appliance interface. That usually happens in biphasic pattern where an initial “burst release” within the first 1–2 h is followed by a slower and more continuous release that can last between 12 h to multiple days [15, 27]. Additionally, no cytotoxic effects have been reported in in vitro settings while still effectively reducing biofilm formation [15]. In comparison, although highly antibacterial, chlorhexidine is known for its possible side effects, such as cross-resistance with antibiotics, discoloration, dysesthesia as well as cytotoxic effects on mucosa [28–30]. There are no indications of a similar adverse outcomes in relation to essential oils. In contrast, essential oil extracts have been reported to modulate antibiotic resistance mechanisms, making it thereby significantly harder for pathogens to develop resistance compared to conventional single-target antibiotics [31–33].

Combining a thermoformable material for dental appliances with an antimicrobial agent could represent a novel approach in the field to reduce microbial colonization and support oral health during long-term treatment with such devices. Moreover, mixtures of essential oils creating a versatile antimicrobial environment on tooth-dental appliance interface have not been studied until now. Therefore, the aim of this study is to investigate the in vitro effect of a cellulose-based thermoformable dental material (NaturAligner, Bottmedical, Switzerland) treated with a solution containing a mixture of essential oils on the biofilm formation of *S. mutans* and of a 3-species biofilm (*Porphyromonas gingivalis*,* Fusobacterium nucleatum*,* Streptococcus sanguinis)*. The null hypothesis is that no difference is detected in biofilm formation whether the materials are loaded with essential oils or not.

## Materials and methods

### Flow chamber experiments of biofilm formation with sandwich system

#### Pre-treatment cellulosed-based thermoformable material

**Fig. 1:**
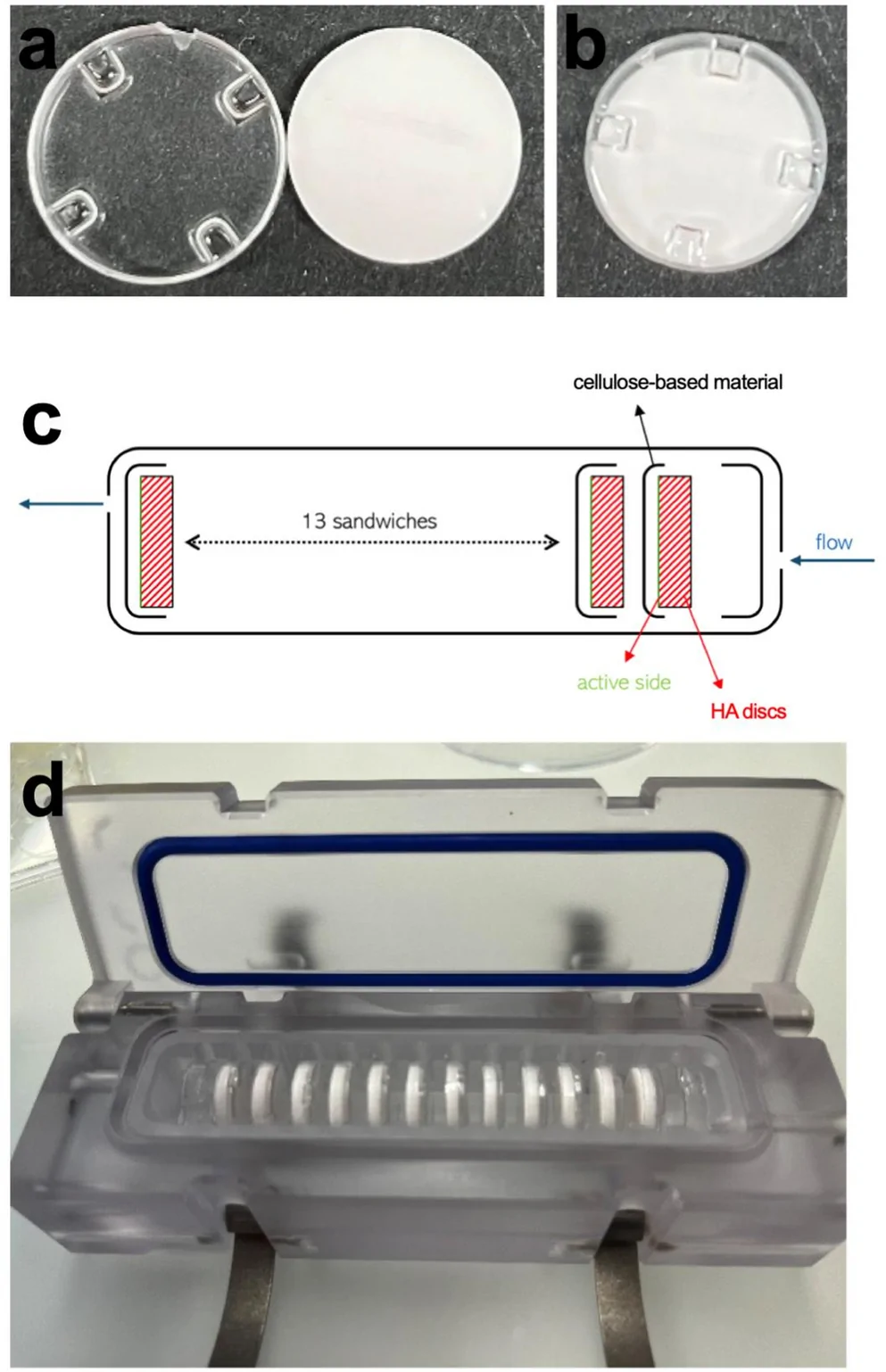
Flow Chamber Set-up: (**a**) cellulose-based material (left) and HA disc (right); (**b**) Saliva-coated HA discs capped over with cellulosed-based material; (**c**) sketch of sandwiches in flow chamber; (**d**) image of a flow chamber with material-disc-sandwiches placed into positions

The cellulose-based multilayered thermoformable material (Naturaligner; Bottmedical; Switzerland) with a thickness of 550 μm was used and prepared as reported previously [15]. Shortly, these thermoformed foils (550 μm, 35 s heated at 220 °C in Biostar; Scheu Dental; Germany) were cut and formed into models as depicted in Fig. 1a (left).

The test group materials (*CS*) were placed into sterile vials with 10 mL of a mixture of essential oils (trans-cinnamaldehyde, (+)-carvone, trans-anethole, (R)-(+)-limonene, eucalyptol, methyl salicylate, carvacrol, and triacetin; CINNA Care Solution, Bottmedical AG, Switzerland) for 1 h on a roller shaker (30 rotations per minute, at RT), *n* = 13 per run (*n* = 39 in a total of three independent experimental repeats). The control group were similarly divided and placed in 10 mL of sterile phosphate-buffered saline (*PBS*) under the same conditions, *n* = 13 per run (*n* = 39 in a total of three independent experimental repeats).

Thereafter, the samples were dried on sterile filter paper before mounted on pre-treated hydroxyapatite (HA; Fig. 1a right) discs as shown in Fig. 1b.

#### Pre-treatment hydroxyapatite discs

In two 24-well plates, 13 HA discs with a thickness of 4 mm (HiMed, Old Bethpage, New York, USA) were used. To simulate a polished tooth surface, the discs were pre-treated by grinding and polishing for 10 s with abrasive papers of increasing grit sizes 1200, 2400, and 4000. After sterilization, each of the HA discs were individually dipped in a sterile pooled saliva (Ethics Committee Northwestern and Central Switzerland, Req-2025-01279) solution containing a buffering solution (67 mM Na_2_HPO_4_ and 67 mM KH_2_PO_4_) at room temperature for 15 min. Each HA disc was immersed in 500 µL of the saliva solution to promote the formation of a protein pellicle on the polished surface (active side of the HA disc). Following the HA disc treatment process, they were then combined with the prepared cellulose-based materials to form sample sandwiches as depicted on Fig. 1b. These sandwiches were then placed into flow chambers (Fig. 1c-d).

#### Preparation of cariogenic biofilms

**Fig. 2:**
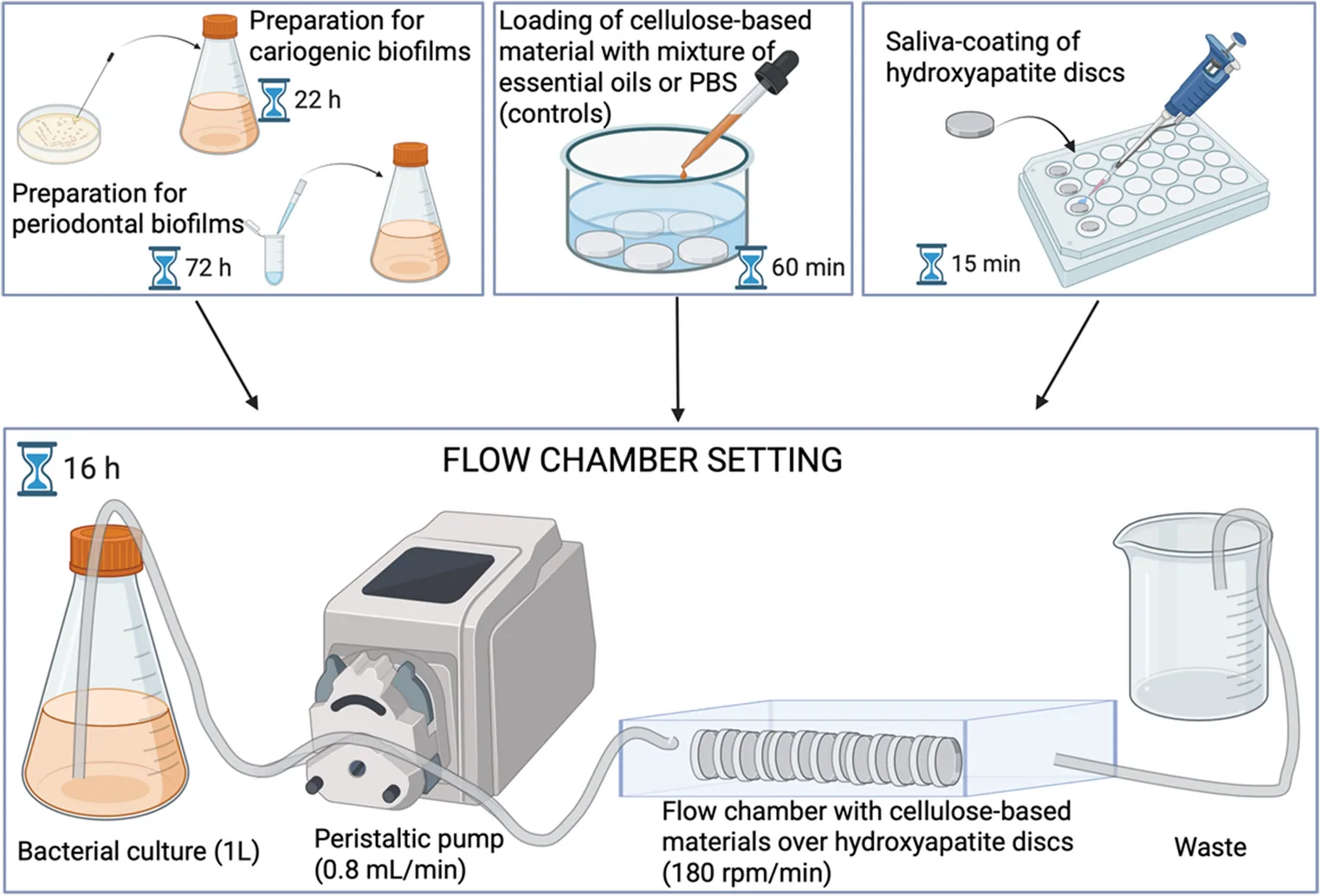
Workflow diagram: Illustration of the preparation (bacteria grown until they reach stationary growth phase) and pre-treatment steps (loading of cellulosed-based materials with mixture of essential oils or PBS for controls, and pellicle formation by saliva treated of hydroxyapatite discs prior to flow chamber) for the flow chamber set-up. The flow chamber experiments last for 16 h; the system is run by a peristaltic pump set to 0.8 rpm/min with the flow chamber placed on a shaker (180 rpm/min) and all bacterial waste is collected to avoid recircling and the possible wash out effects of essential oils to the bacterial culture (Created in BioRender. Astasov-Frauenhoffer, M. (2026) https://BioRender.com)

The overall workflow is depicted in Fig. 2. To prepare cariogenic biofilms, the *S. mutans* strain ATCC 25,175 was utilized. *S. mutans* was grown by spreading stock solution onto blood agar (BA) plates and incubating at 37 °C in an aerobic CO_2_ environment for 72 h. Thereafter, around 30 colonies were selected and transferred into 4 × 250 ml Todd Hewitt (TH) medium supplemented with 0.5% sucrose. The cultures were then incubated for a further 22 h at 37 °C under aerobic conditions. Then the overnight cultures were divided into 50 mL Falcon tubes, sonicated for 30 s at 80% amplitude and centrifuged at 9’200 rpm for 5 min at RT to separate the bacteria from the nutrient medium. A total of 10 bacterial pellets were then resuspended in 775 mL of pre-warmed simulated body fluid (SBF; 37 °C, pH 7.2), which consisted of NaCl (8 g/L), NaHCO_3_ (0.353 g/L), KCl (0.224 g/L), K_2_HPO_4_ × 3H_2_O (0.228 g/L), MgCl_2_ × 6H_2_O (0.305 g/L), CaCl_2_ × 2H_2_O (0.368 g/L), Na_2_SO_4_ (0.071 g/L), Tris (6.055 g/L), and 6% HCl dissolved in 1 L ultra-pure water [34]. The suspension was supplemented with 100 mL of TH medium (final concentration of 10%) and 125 mL of 40% sucrose (final concentration of 5%). Altogether, 2 L of bacterial culture was prepared: 1 L for the test group (*CS*) and 1 L for the control group (*PBS*).

#### Preparation of periodontal biofilms

For the periodontal biofilms, a total of three bacterial strains were utilized. These strains consisted of *Porphyromonas gingivalis* (ATCC 33277), *Fusobacterium nucleatum* (ATCC 10953), and *Streptococcus sanguinis* (DSM 20068).

Using previously frozen aliquots, precultures of *P. gingivalis* and *F. nucleatum* were prepared in Thio+ (thioglycolate broth from Sigma Aldrich, Buchs, Switzerland, supplemented with hemin (5 mg/L) and menadione (0.5 mg/L)) and incubated anaerobically at 37 °C for at least 72 h. The *S. sanguinis* precultures were grown in brain-heart infusion (BHI; Sigma Aldrich, Buchs, Switzerland) and incubated at 37 °C for 12–17 h in an aerobic environment, then sonicated for 30 s at 80% amplitude.

The bacterial cultures were combined in 50 mL Falcon tubes and centrifuged under the same conditions as described above. Bacterial pellets were then resuspended in 867.75 mL of pre-warmed SBF (37 °C) supplemented with 97.5 ml Thio+ (final concentration of 10%) and 9.75 mL of 20% sucrose (final concentration of 0.5%) under anaerobic conditions.

Altogether, 2 L of bacterial culture was prepared: 1 L for the test group (*CS*) and 1 L for the control group (*PBS*).

#### Control of bacterial growth

In order to determine the bacterial concentration at t = 0, serial dilutions of the bacterial culture were made and 10 µL drops were placed onto previously prepared Columbia blood agar (BA) plates (Columbia-Agar Base from Becton Dickson, Basel, Switzerland, supplemented with human blood (50 mg/L), menadione (0.5 mg/L), and hemin (5 mL/L) poured into standard Petri dishes). These plates were then incubated aerobically for streptococcal species and anaerobically for periodontal species at 37 °C, for 3 d or 7 d, respectively, followed by counting the colony forming units (CFU).

#### 16 h flow chamber biofilm formation

After preparation, the flow chamber system was complied. It consisted of a bacterial suspension, a flow chamber containing a group of samples (*CS* or *PBS*) on a shaker (set at 180 rpm to maintain a homogeneous solution), a peristaltic pump operating at 0.8 mL/min to imitate saliva flow, and a waste collection bottle for the used bacterial suspension. Circulating bacteria were allowed to adhere to the materials in the flow chambers at 37 °C for 16 h in either an aerobic (cariogenic biofilms) or anaerobic (periodontal biofilms) environment.

### Evaluation of biofilm formation

To assess biofilm formation on HA discs and material, the sample sandwiches were carefully removed from the flow chamber after 16 h, separated and dipped in 0.9% NaCl, and air dried on sterile filter paper. The dried biofilms were stained with 300 µL of 0.1% crystal violet solution (CV) for 10 min. Excess unbound dye was then removed by dipping the samples in distilled water. After air drying, the samples were destained by using 1 mL of 25% acetic acid, and the absorbance was measured at 595 nm using a spectrophotometer (BioTek Synergy 2, BioTek Instruments AG, Lucerne, Switzerland), with 800 µL of pure 25% acetic acid used as a blank.

### Determination of growth inhibition and antimicrobial activity by isothermal microcalorimetry (IMC)

**Fig. 3:**
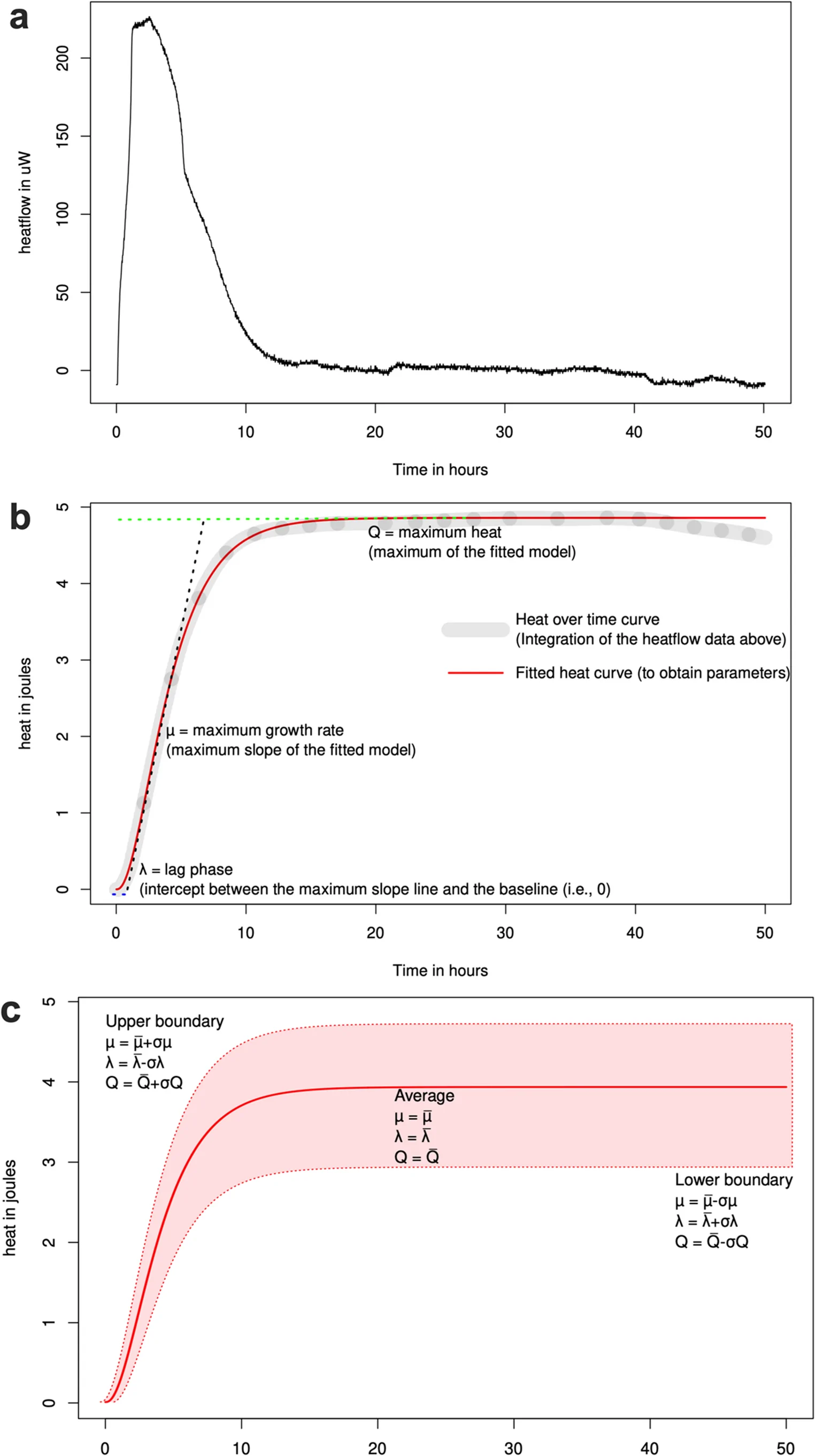
IMC data analysis. The raw heat flow curves obtained by IMC (**a**) are integrated to obtain total heat over time curves similar to a growth curve (**b**). These data can be fitted using the modified Gompertz model as rearranged by Zwietering et al. 1990 and limited to use the following parameters: µmax the maximum growth rate and λ the duration of the lag phase, Qt the heat produced at time t, and Qmax the maximum heat (**c**) [35, 36]

In order to determine the antimicrobial activity of the mixture of essential oils against *Porphyromonas gingivalis* (ATCC 33277) and *Fusobacterium nucleatum* (ATCC 10953), IMC was used [15, 21]. Before use, precultures were prepared as described above. In an anaerobic environment, 10 µL of the preculture was placed on a disc of thermoformable material (NaturAligner, Bottmedical, Switzerland) loaded with essential oils. The inoculated side of the disc was placed facing solid BA prepared in 10 mL vials. Light pressure was applied to the back of the disc until the liquid was evenly distributed across the surface between the disc and the agar. The vials were sealed and placed in a calorimeter (TAM air). After placing the vials in the calorimeter and allowing 1 h of thermal equilibration, the heat flow was measured. Negative controls were prepared using discs exposed to PBS for 1 h. At the end of the experiment, data was collected and transformed into an ASCII file. The raw heat flow curves were integrated to obtain total heat over time curves similar to a growth curve. These data were then fitted using the modified Gompertz model as rearranged by Zwietering et al. 1990 and limited to use the following parameters: µmax the maximum growth rate and λ the duration of the lag phase, Qt the heat produced at time t, and Qmax the maximum heat [35, 36] as also illustrated in Fig. 3.

### Statistical analysis

To assess statistical differences between groups, the distribution of results was first analyzed, and the group of data have been tested for significant outliers. An unpaired t-test was performed to compare samples exposed to PBS with those exposed to CS. This analysis was performed for both HA discs and material. Statistical difference was set at *p* < 0.05 (GraphPad Prism version 10.0.0 for Mac, GraphPad Software, Boston, Massachusetts USA, www.graphpad.com). In addition, biofilm reduction was calculated for samples treated with CS compared to PBS controls.

## Results

### Biofilm formation CV staining

**Fig. 4:**
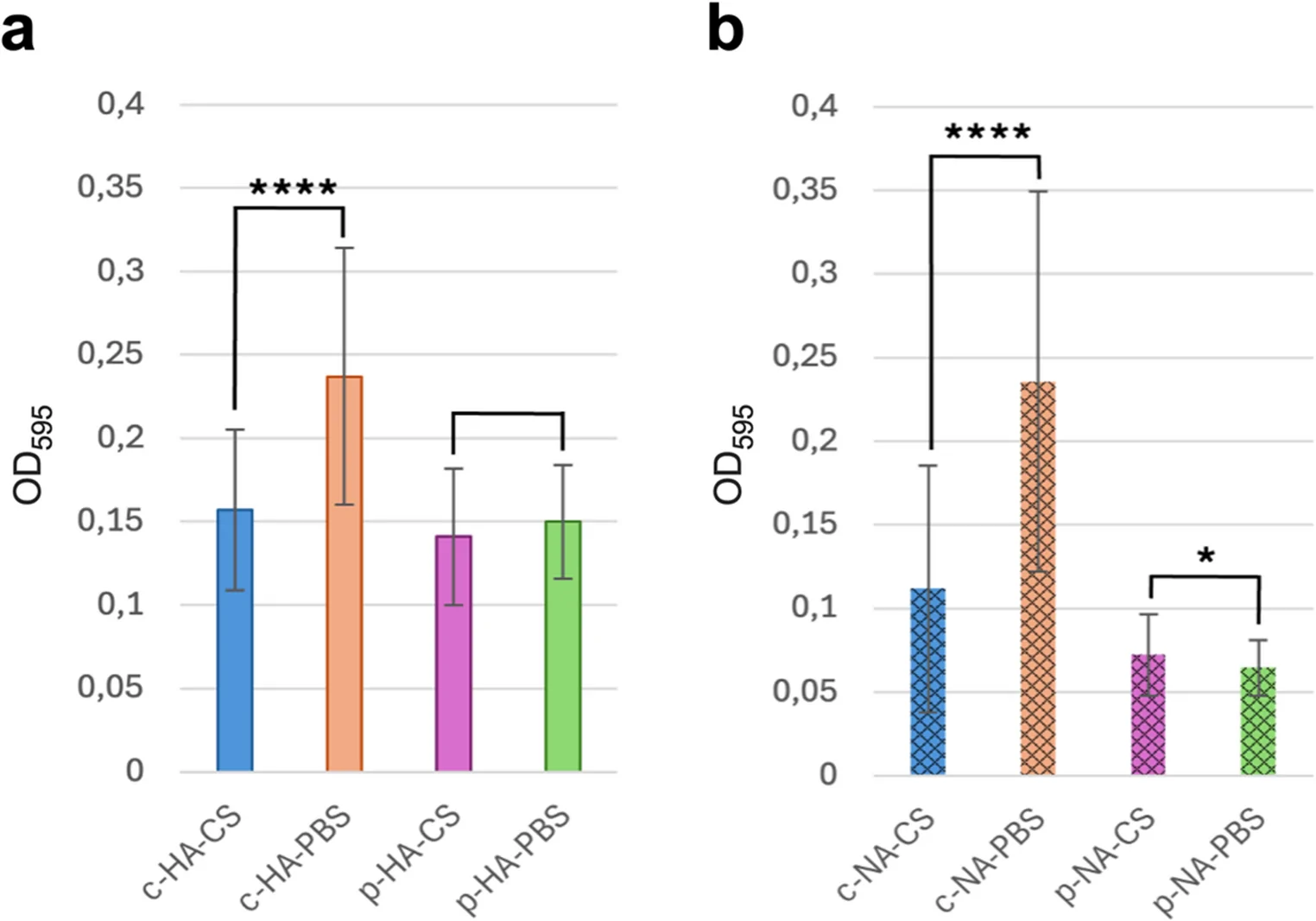
Quantitative analysis of biofilm formation on the interface between (**a**) hydroxyapatite (HA) and (**b**) cellulose-based material (NA), following 1 h exposure to essential oil mixture (CINNA Care Solution (CS)) compared to control (PBS). Two different biofilms were assessed (“c”; *S. mutans* and periodontal (“p”; 3-species biofilm). Biofilm was allowed to form for 16 h and was quantified by CV staining at OD595. Bars represent mean biofilm values; error bars show standard deviation. Significance is indicated by asterisks (*p < 0.05; ****p < 0.001)

In Fig. 4, relative biofilm formation of cariogenic (“c”; *S. mutans)* and periodontal (“p”; 3-species biofilm) biofilms is presented. Figure 4a shows results for HA discs and Fig. 4b for cellulose-based material. The results demonstrate a visible antimicrobial effect of the mixture of essential oils added to material for the groups with cariogenic biofilm (c-HA / c-NA). Cellulose-based material with essential oils led to a reduction of 34% of cariogenic biofilm on the surface of HA discs compared to control, while the reduction on material was even higher with a value of 52.5%.

However, there was no significant effect of the antimicrobial on reducing periodontal biofilm formation. Only a small reduction was observed on HA discs (6.3%) but not on the cellulose-based essential-oil-coated material where a slight increase of biofilm formation could be measured (-12%).

### Isothermal microcalorimetry (IMC) for periodontal pathogens measured as single species

**Fig. 5:**
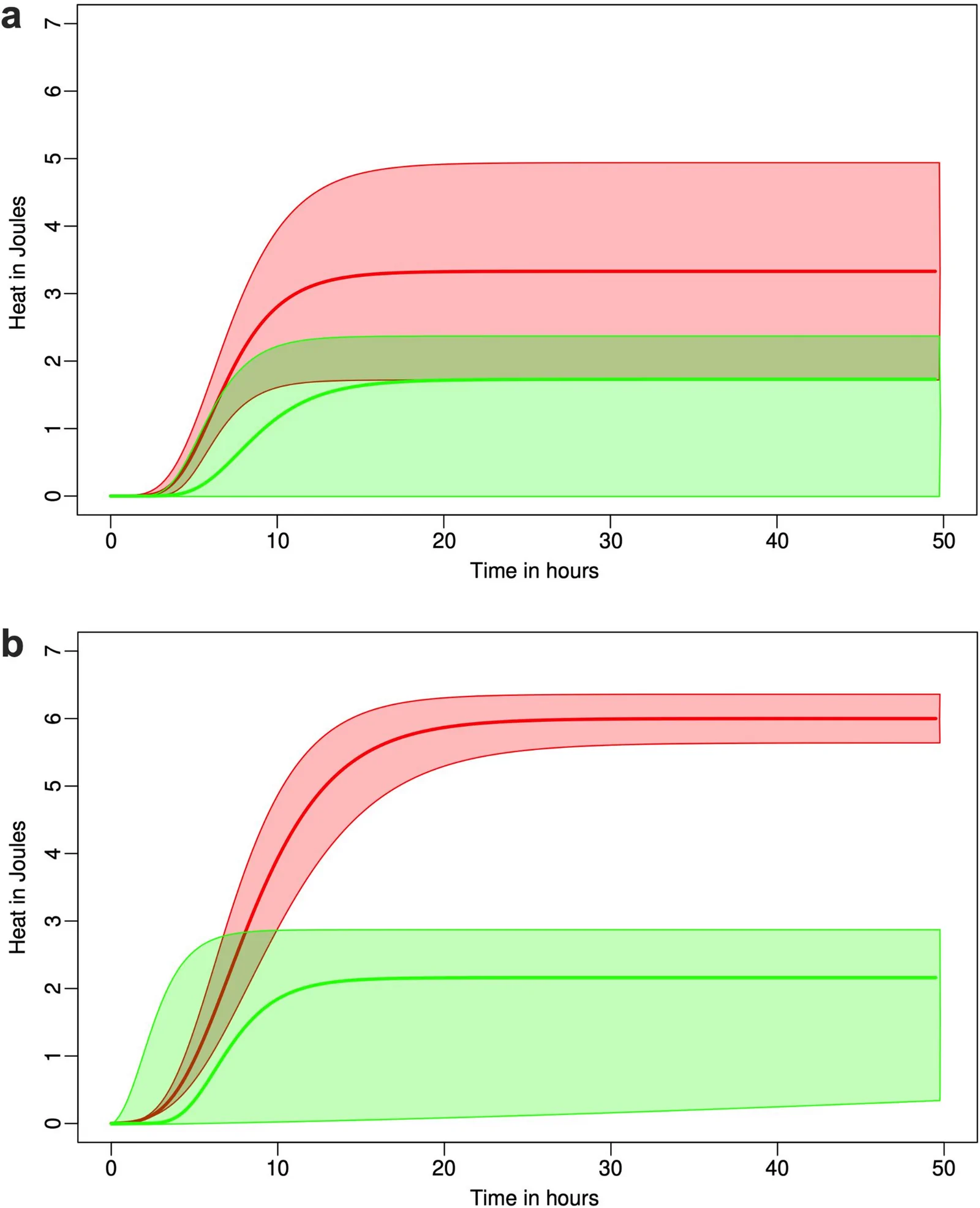
IMC total heat calculated from heat flow for F. *nucleatum* (**a**) and *P. gingivalis* (**b**) in incubation of material loaded either with CS (green line indicating the mean value and area marking upper and lower boundary) or with PBS (red line indicating the mean value for mean heat and area marking upper and lower boundary)

Single-species analysis of IMC revealed that the periodontal pathogens react differently to the essential oil mixture used in this study. For both pathogens, growth rate (µ) and total heat produced (Q) were decreased. For both pathogens also the lag phase (λ) was increased indicating that a small portion of the inoculum might have been inhibited. Overall *P. gingivalis* (Fig. 5b) seemed more sensitive than *F. nucleatum* (Fig. 5a). However, these results suggest that efficacy shown for the single-species here, is not more in mixed species biofilm as shown above in flow chamber experiments; even though it is having an inhibitory effect on streptococci as shown in previous studies [15], it only minimally inhibits the growth and biofilm formation of the periodontal pathogens especially when tested in mixed cultures (Fig. 5).

## Discussion

The management of oral biofilm formation on intraoral appliances remains challenging. The surface of orthodontic devices, clear aligners, retainers, nightguards, and splints provides microenvironments that facilitates biofilm accumulation leading to increasing risk of caries and periodontal diseases during prolonged wear. Recent studies confirm that conventional thermoplastic materials such as PETG, PMMA, and 3D-printed polymers support robust biofilm formation within 24–120 h of exposure [4, 6, 37]. This highlights the need for innovative strategies that can assist to reduce bacterial adherence at the tooth-appliance interface.

In this study, loading a cellulose-based thermoformable material with an antimicrobial solution containing essential oils led to a significant reduction in *S. mutans* biofilm formation under dynamic flow conditions. This effect is clinically relevant given the central role of *S. mutans* in cariogenic biofilms and the findings align with recent data showing that essential oil nanoemulsions containing cinnamon as well as other plant-derived essential oils can inhibit oral biofilm formation, often through mechanisms involving disruption of cell membranes, inhibition of glycosyltransferase activity, suppression of acid production or interference with quorum sensing [38–41]. Furthermore, the ability to reload the cellulose-based appliance with essential oils at regular (daily) intervals provides a practical approach to maintain antimicrobial activity throughout the treatment without permanent chemical modifications or altering the mechanical properties of the material [15].

While *P. gingivalis* alone showed some sensitivity to the antimicrobial mixture used, there was no significant inhibition of multi-species biofilm under flow conditions. However, these findings are consistent with recent studies showing that essential oils often demonstrate a strain- and microbiome community-dependent efficacy and their impact is more pronounced against cariogenic streptococci rather than mature multispecies or anaerobic periodontal communities [8, 38, 42]. Still, there is no harmful effect, as the cellulose-based material remains safe towards human gingival fibroblasts and the slow release kinetics of the bioactive molecules of the essential oils allow the application to work at low concentrations without affecting the surrounding tissue as has been reported previously [15]. Thus, this indicates further that early biofilm formation can easily controlled by the suggested approach and with good compliance by the patient the shift from healthy to biofilm-driven diseases could be withheld.

The antimicrobial activity demonstrated in this study is in line with promising in vivo studies using essential oils or single bioactive molecules purified from essential oils. Cinnamon essential oil, the main component of the mixture tested in the present study, has been shown to reduce plaque and gingival index as significantly as chlorhexidine gluconate (0.2%) [21]. Its efficacy against oral pathogens like *S. mutans*, *S. mitis* and *S. epidermidis* has been described [7, 28]. Moreover, some research has also shown that cinnamaldehyde inhibits the biofilm formation of *P. gingivalis* and should therefore be an antimicrobial agent against periodontal disease [16]. This was not confirmed by the present investigation as the antimicrobial effect was significantly diminished in the three-species setting. Moreover, here a flow chamber model with open circulation of the bacterial suspension and therefore sheer stress on adherent cells was used, while Wang et al. presented a closed/static study model without flow conditions and sheer stress. Periasamy et al. (2009) showed that although the initial attachment of *P. gingivalis* at 4 h after tooth cleaning to saliva-coated surface in the flow cell took place, the bacteria failed to establish a matured biofilm over 14 h due to the dynamics of the flow, with metabolically stressed cells unable to maintain adherence under flow conditions [43]. This may explain the overall reduced biofilm formation in the periodontal biofilm PBS group compared to cariogenic biofilm PBS group. Nevertheless, static studies do not represent the continuous flow conditions in the oral cavity. Therefore, our flow-based study model provides a more accurate representation of in vivo conditions.

Finally, a limitation of the present study lies in the number of bacterial strains studied. More than 700 species live in the oral cavity [44]. This study cannot assess the antimicrobial effect of all the combinations of bacteria, and it does not include the interspecies interactions with more complex extracellular matrix; thus, the differences in susceptibility among species may reduce the overall response, and the effect in an in vivo setting might be less profound. However, this study does give a first insight in case the diseases progress and pathogenic strains dominate the biofilms, what kind of effect could be expected. Furthermore, this study used an in vitro setting, and clinical trials need be conducted to study the in vivo effect of cellulose-based material loaded with antimicrobial agents. Despite this, these results are promising, as this represents the first model of its kind to allow biofilm formation under flow conditions in a bioreactor, consisting of setting mimicking appliances covering tooth surfaces to study biofilm formation on the interface of both surfaces simultaneously.

Nevertheless, the previous [15, 21] and present work shows that a cellulose-based thermoformable dental material can be treated and loaded with essential oils to achieve substantial reductions in cariogenic biofilm formation under clinically relevant conditions, without compromising mechanical performance or cytocompatibility. This supports the potential combined use (thermoformable material with regular application of essential oils) as a platform technology for next-generation intraoral appliances, including, aligners, retainers, splints, that combine mechanical function with active infection control.

## Data Availability

Upon publication raw data will be accessible on Zenodo repository with the identifier: 10.5281/zenodo.20749334.
